# The In-Vitro Activity of a Cold Atmospheric Plasma Device Utilizing Ambient Air against Bacteria and Biofilms Associated with Periodontal or Peri-Implant Diseases

**DOI:** 10.3390/antibiotics11060752

**Published:** 2022-05-31

**Authors:** Gert Jungbauer, Leandro Favaro, Steffen Müller, Anton Sculean, Sigrun Eick

**Affiliations:** 1 Department of Periodontology, School of Dental Medicine, University of Bern, 3010 Bern, Switzerland; leandro@favaro.li (L.F.); anton.sculean@unibe.ch (A.S.); sigrun.eick@unibe.ch (S.E.); 2 Independent Researcher, Private Dental Practice, 94315 Straubing, Germany; 3 Department of Cranio-Maxillofacial Surgery, Hospital of the University of Regensburg, 93053 Regensburg, Germany; steffen.mueller@ukr.de

**Keywords:** cold atmospheric plasma, non-thermal, atmospheric pressure, periopathogens, *Porphyromonas gingivalis*, periodontal, peri-implant, titanium

## Abstract

Due to its antimicrobial and healing-promoting effects, the application of cold atmospheric plasma (CAP) appears to be a promising modality in various fields of general medicine and dentistry. The aim of the present study was to evaluate the antibacterial and anti-biofilm activity of a handheld device utilizing ambient air for plasma generation. Suspensions of 11 oral bacteria (among them *Fusobacterium nucleatum*, *Porphyromonas gingivalis*, *Parvimonas micra*, *Streptococcus gordonii*, and *Tannerella forsythia*) were exposed to CAP for 10, 30, 60, and 120 s. Before and after treatment, colony forming unit (CFU) counts were determined. Then, 12-species biofilms were cultured on dentin and titanium specimens, and CAP was applied for 30, 60, and 120 s before quantifying CFU counts, biofilm mass, and metabolic activity. A reduction of ≥3 log_10_ CFU, was found for ten out of the eleven tested species at 30 s (except for *T. forsythia*) and for all species at 60 s. For biofilm grown on dentin and titanium specimens, the log_10_ reductions were 2.43 log_10_ CFU/specimen and by about 4 log_10_ CFU/specimen after 120 s of CAP. The CAP application did not reduce the biomass significantly, the metabolic activity of the biofilms on dentin and titanium decreased by 98% and 95% after 120 s of CAP. An application of 120 s of CAP had no cytotoxic effect on gingival fibroblasts and significantly increased the adhesion of gingival fibroblasts to the titanium surface. These results are promising and underline the potential of CAP for implementation in periodontal and peri-implantitis therapy.

## 1. Introduction

According to epidemiologic data, the prevalence of periodontitis is very high, about 10% of the global population is affected by a severe form of the disease [1]. Periodontitis is associated with an impairment of the host response, an imbalance of oral microbiota leading to an overgrowth of putative pathogens (e.g., *Porphyromonas gingivalis*, *Tannerella forsythia*, and *Filifactor alocis*), disruption of tissue homeostasis, and periodontal destruction [2,3,4,5]. Moreover, the prevalence of peri-implantitis is high, a recent systematic review reported a weighted mean prevalence of 22% across Europe and North and South America [6]. Similar to periodontitis, the microbiota associated with peri-implantitis is dominated by well-known periodontal pathogens, such as *P. gingivalis*, *T. forsythia*, and *Treponema denticola* which suggests a similar biofilm-induced and host-mediated pathogenesis and therefore a similar anti-infective treatment of peri-implantitis and periodontitis [7], although there may be some differences in the specific biofilm formation and final composition due to surface properties and corrosion of titanium [8]. 

An essential part of periodontal and peri-implant therapy is the mechanical removal of the subgingival or peri-implant biofilm. In many cases, the sole mechanical debridement is not sufficient and adjunctive measures such as antiseptics or antibiotics have to be applied [9,10]. In particular, the high consumption of antibiotics in the therapy of periodontitis and especially peri-implantitis [11,12] necessitates the search for alternatives. One possible option might be the adjunctive application of cold atmospheric plasma (CAP). 

Plasma is defined as the fourth state of matter, besides solid, liquid, and gaseous. It can be generated by ionizing gas in an electric field between two electrodes separated by a dielectric barrier [13]. These plasma-generating devices can be indirect devices such as jet or surface dielectric barrier discharge (SDBD) devices. Direct plasma devices use the treated surface as grounded electrode, and the plasma can be ignited utilizing ambient air, such as a corona discharge or volume dielectric barrier discharge (VDBD) [13]. Commercially available devices are, among others, the direct DBD device plasma ONE (plasma Medical Systems, Nassau, Germany), the indirect DBD PlasmaDerm (Cinogy, Dulderstadt, Germany), and the jet device kINPen med (neoplas med, Greifswald, Germany). The application of CAP has emerged in the medical field, including the dental field, in the recent decade and the areas of application are manifold, e.g., treatment of chronic and acute wounds, regenerative medicine [14], orthopedic and vascular surgery, ophthalmology [15], and oncology [16]. In dentistry, CAP application might be of interest for many indications, such as instrument sterilization, disinfection of cavities after the removal of dental caries, teeth bleaching, increase of the bonding of adhesive restorations and wound healing, treat *Candida* infections and remove biofilm [17]. In terms of an anti-biofilm effect, the use of CAP is discussed in endodontics [18] and non-surgical periodontal therapy [19], but in particular in implantology and the therapy of peri-implant diseases [20]. In animal studies, the combination of mechanical debridement and adjunctive CAP application on contaminated dental implants led to superior periodontal outcomes such as alveolar or peri-implant bone level, less inflammation, and a decreased detection rate of periopathogens [21,22,23]. In a recent split mouth clinical study, periodontitis patients with adjunctive application of CAP showed a better clinical and microbiological outcome compared to the controls without CAP [19].

The antimicrobial effect of CAP is primarily based on the generation of reactive oxygen (ROS) and nitrogen species (RNS), accompanied by electrons, ionized particles, electric fields, and radiation [24]. In general, the generation of ROS and RNS affects various microbial structures. The cell wall could be etched and the membrane damaged by disruption and lipid peroxidation. The bacterial DNA and RNA could be compromised by oxidative damage, base modification, and strand breaks, and proteins may become unfolded or modified [24]. The antimicrobial effects are dependent on many factors including the device parameters and the experimental setup, but also the treated pathogens [25]. Most available studies investigating the antimicrobial effect of CAP used argon or helium with or without an admixture of oxygen as working gases. In terms of clinical implementation, gas cylinders are needed which makes the application more complicated concerning administration and occupational safety [25]. 

Therefore, devices operating with ambient air might be advantageous. Every manufacturer uses his own configuration to create an electric field to induce plasma generation. The recent advancement of a handheld plasma device series, originally designed for non-medical surface modification, uses the underlying principle of a piezoelectric direct discharge (PDD). The heart of such a device consists of a piezoelectric transformer. A high conversion ratio and thus, high electric field strengths are achieved with Rosen-type [26] transformers where a resonant longitudinal wave is excited in a piezoceramic rod-shaped element (e.g., PZT, lead zirconium titanate) via the electrical excitation. To work with low input voltages, the first half of the element consists of multilayers polarized perpendicular to the applied electric field, and the second half of the rod consists of a region of the piezoelectric material polarized parallel to the oscillation axis. In resonance, the entire rod is deformed at high frequency (25–100 kHz) in the micrometer range, and the secondary side generates a high voltage with a transformation ratio of up to 10,000 with respect to the input voltage due to the inverse piezoelectric effect. This principle achieves a sufficiently high voltage to ignite corona type plasma in ambient air [27]. The device was originally developed to optimize manufacturing processes in terms of coating and bonding. In the medical and dental field, studies with plasma generated by this PDD technology showed an antibacterial effect against single species biofilms of *Staphylococcus aureus*, *Listeria monocytogenes*, and *Salmonella* [28,29,30] and increased adhesion of host tissue cells on titanium surfaces [28,31]. At present, there is no data on the potential activity of CAP using this direct handheld PDD device on oral planktonic bacteria and on multi-species biofilms associated with periodontal and peri-implant diseases established on titanium or dentin surfaces.

The aim of this in-vitro study was to analyze the activity of CAP using a direct handheld PDD device against planktonic oral bacteria and periodontal/peri-implant multi-species biofilms and on adhesion of oral fibroblasts on dentin and titanium specimens. Exposure times applicable in a dental practice were used in the experimental designs.

## 2. Materials and Methods

### 2.1. Plasma Device

**Figure 1:**
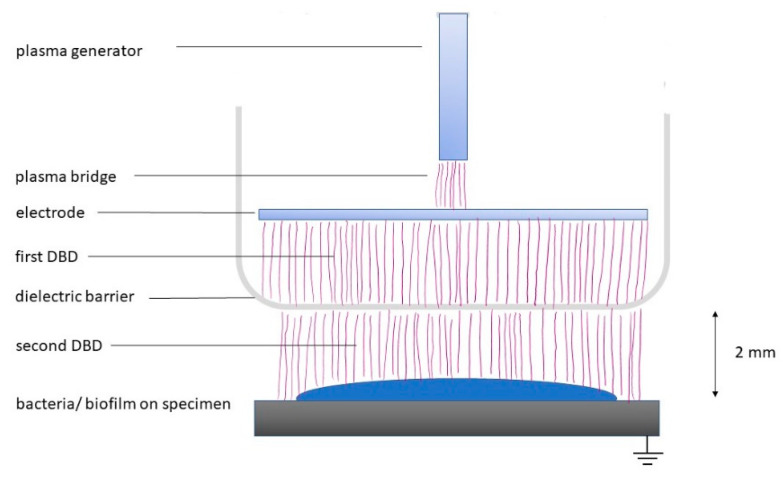
Experimental set-up for the effect on planktonic bacteria and biofilms; DBD: dielectric barrier discharge.

For the experiments, the commercially available plasma PDD device (piezobrush^®^ PZ3; Relyon Plasma, Regensburg, Germany) was used, operating at about 50 kHz with a power of about 8 W. The device was originally designed for surface treatments in the industrial and handcraft sector. The so-called near-field nozzle with a dielectric barrier cap was used (Figure 1). The treated surface (polystyrene, dentin, or titanium) acted as the grounded electrode. Here, the PDD device was fixed at a working distance of 2 mm between the nozzle and the surface to be treated (Figure 1). The respective treatment time was set on the device (countdown) and the control signal in the display indicated the ignition of the plasma.

### 2.2. Bacteria

The following 12 bacterial strains were used in the experiments: *Streptococcus gordonii* ATCC 10558, *Actinomyces naeslundii* ATCC 12104 (both species represent early colonizers), *Capnocytophaga gingivalis* ATCC 33624, *Campylobacter rectus* ATCC 33238, *Eikenella corrodens* ATCC 23834, *Fusobacterium nucleatum* ATCC 25586, *Prevotella intermedia* ATCC 25611, *Parvimonas micra* ATCC 33270 (representing bacteria functioning as bridges between early and late colonizers), *Porphyromonas gingivalis* ATTC 33277, *Filifactor alocis* ATCC 33238, *Tannerella forsythia* ATCC 43037, and *Treponema denticola* ATCC 35405 (late colonizers, being strongly associated with periodontal disease).

The strains were maintained on tryptic soy agar (TSA) plates (Oxoid, Basingstoke, UK) with 5% sheep blood under anaerobic conditions (*S*. *gordonii* with 5% CO_2_) at 37 °C. *T*. *denticola* was maintained in modified mycoplasma broth (BD, Franklin Lake, NJ, USA) with the addition of 1 g/mL cysteine, 1 mg/mL glucose, 400 μg/mL niacinamide, 150 μg/mL spermine tetrahydrochloride, 20 μg/mL Na isobutyrate, and 5 μg/mL cocarboxylase under anaerobic conditions at 37 °C.

### 2.3. Activity on Planktonic Bacteria

For the experiments, the bacterial strains (except for *T*. *denticola*) were suspended in 0.9% *w*/*v* NaCl, and adjusted to the McFarland Standard 0.5 (1.5 × 10^8^ CFU/mL). One milliliter of each suspension was placed in an Eppendorf tube and centrifuged for 2 min at 7000× *g* at 20°C. The supernatant was discarded, and the sediment was resuspended in 250 µL 0.9% *w*/*v* NaCl and pipetted in a deepening of a polystyrene slice. After that, CAP was applied for 10, 30, 60, and 120 s. The order of application time was changed in each experimental series to avoid an influence of the suspension media on the bacterial cell viability as a systematic error. Subsequently, the bacteria were resuspended with 1 ml of 0.9% NaCl, serially diluted, and aliquots were plated on agar plates. After an incubation time for 8 d (*S. gordonii* 24 h), the colony forming units (CFU) were counted using a colony counter (aCOLyte, Synoptics Ltd., Cambridge, UK). 

### 2.4. Dentin and Titanium Specimen Preparation

Dentin specimens were prepared from extracted human teeth. The volunteers were informed about the use of their extracted teeth in research, and their oral consent was obtained. According to the guidelines, no previous approval from the Cantonal Ethical Committee Bern (KEK) was necessary as the teeth were categorized as “irreversibly anonymized”. Dentin slices were trimmed and smoothed to a size of 5 × 5 × 1 mm (length × width × height) using diamond burs and sandpaper. Before using, slices were placed in water and autoclaved at 121 °C for 20 min. Titanium disks with a sandblasted and etched surface and with a diameter of 5 mm were provided by Straumann (Basel, Switzerland). 

### 2.5. Multi-Species Biofilm

For analyzing the effect of CAP on a multi-species biofilm, all 12-species were included. The protocol for biofilm is a well-established model in our laboratory, the different bacterial species were counted between 5 and 7 log_10_ CFU each [32]. The mixture of the different bacteria was always checked by the presence of the different colonies typical for the species on TSA plates supplemented with 5% sheep blood. The dentin or titanium specimens were placed in the wells of 24-well plates and pretreated with 20 µL of protein solution (1.5% bovine serum albumin) for 30 min. Meanwhile, the inoculum was prepared by adjusting bacterial suspensions to the McFarland Standard 0.5. One part *S*. *gordonii* was mixed with two parts of *A*. *naeslundii* and four parts of the other species. This mixture was added to the nutrient broth (Wilkins-Chalgren-broth (Oxoid, Basingstoke, UK) with 10 µg/mL β-NAD (Merck KGaA, Darmstadt, Germany) in a ratio of 1:9 before 1 mL was pipetted per well. The biofilm was incubated under anaerobic conditions (85% N_2_, 10% H_2_, 5% CO_2_) at 37 °C in an anaerobic chamber (Don Whitley Scientific Limited, West Yorkshire, UK). The culture medium was replaced after 48 h and selected species (*P. gingivalis, T. forsythia* and *T. denticola*) were added again. After 3.5 days, the nutrient broth was removed, and the biofilms were carefully washed with 0.9% *w*/*v* NaCl to remove free floating bacteria. Then, the specimens with the biofilms were placed in a 12-well-plate and CAP was applied for 30, 60, and 120 s. Afterward, the specimens were transferred into tubes containing 1 mL 0.9% *w*/*v* NaCl. The biofilm was removed by extensive ultrasonication for 5 min. The suspension was handled as described above. 

Thereafter, we determined the following variables:

(a) The total numbers of CFU on TSA plates were counted after an incubation time of 8 d.
(b) Quantification of the biofilm was made after staining with crystal violet according to a recently published protocol [33,34]. After CAP treatment, the 100 µL of the biofilm suspension was transferred to a 96-well plate and fixed for 1 hour at 60 °C. For staining, 50 μL of 0.06% (*w*/*v*) crystal violet (Merck KGaA, Darmstadt, Germany) was added per well. The biofilm mixture was incubated for 10 min at room temperature and dissolved with 200 μL of 30% acetic acid. The plate was read at 600 nm by a microplate reader (ELx808, BioTek Instruments, Winooski, VT, USA).
(c) The metabolic activity of the biofilm was assessed using resazurin as a redox indicator. After transferring 100 µL of the biofilm suspension to another 96-well-plate, 100 μL of nutrient broth containing 0.06 µL resazurin solution (alamarBlue^®^ reagent, Thermo Fisher Scientific Inc., Waltham, MA, USA) was added per well. After 1 h of incubation, the 96-well plate was measured at 570 nm against 600 nm using (ELx808, BioTek Instruments, Winooski, VT, USA).

In another series of experiments, dentin or titanium specimens were first exposed to 120 s CAP, before the biofilms were cultured in the same way as before. The time of incubation was 4 h and 24 h. Analyses were made as before after 24 h of incubation; after 4 h only CFU counts were determined.

### 2.6. Adhesion of Oral Fibroblasts

Human gingival fibroblasts were anonymously collected from systemically healthy patients during regular surgical procedures following written informed consent. This procedure is approved by the Ethics Committee of the University of Bern. The fibroblasts were grown to confluence in T-25 cell culture flasks containing DMEM (Life Technologies/Invitrogen, Paisley, UK) with 10% fetal calf serum (Life Technologies/Invitrogen). Fibroblasts in the third–fifth passage were used. Dentin and titanium specimens were placed into 24-well plates and were left untreated or irradiated with CAP for 120 s. Thereafter, gingival fibroblasts in culture medium at a density of 10,000 cells per well were added and incubated at 37 °C with 5% CO_2_. 

After 72 h of incubation, the gingival fibroblasts were fixed and stained with DAPI (Roche Diagnostics, Mannheim, Germany) and counted using a fluorescence microscope (Olympus BX51, Tokyo, Japan). Eight fields of 1 mm^2^ each were counted and one mean value was calculated per specimen. The number of cells was counted using the ImageJ software and the cell counter plug-in.

### 2.7. Experiments on Toxicity

For the cytotoxicity assay, gingival fibroblasts were pre-cultivated to confluence on glass cover slips. The cover slips were transferred to 12-well plates and the cell cultures were exposed to CAP for 10, 30, 60, and 120 s. Thereafter, 0.2% trypan blue staining was added, and the percent of dead cells was counted by using an inverse microscope. Then, the ratio vital cells/total cells was calculated. 

Dentin and titanium specimens were irradiated with CAP for up to 120 s at a nozzle-to-specimen distance of 2 mm and the temperature was measured by infrared thermography (TIM 160, Micro-Epsilon, Ortenburg, Germany). For calculating the temperature, the emissivity coefficients ε = 0.95 for dentin and ε = 0.50 for titanium [35] were used. 

### 2.8. Statistical Analysis

The experiments using planktonic bacteria were performed as independent quadruplicates. In all biofilm experiments, each six independent biological samples entered statistical analysis. The bacterial counts were recorded as log_10_ CFU. In presentation, the arbitrary units of the activity and the mass data of the biofilm were related to the means of the untreated controls. The biofilm data was compared by applying one-way analysis of variance (ANOVA) with a post-hoc comparison using Bonferroni correction. 

Regarding adhesion of gingival fibroblasts, eight independent results entered the analysis, the Students t-test was used. The statistical analysis was performed with SPSS 24 (IBM, Armonk, NY, USA). 

## 3. Results

### 3.1. Bactericidal Effect on Oral Bacteria

First, the bactericidal activity on planktonic bacteria was quantified. A time-dependent effect was visible. Plasma application of 10 s reduced the CFU counts by 0.69 log_10_ CFU in mean (0.12 log_10_ for *A. naeslundii* up to 1.12 log_10_ CFU for *S. gordonii*). After 30 s of application, the CFU counts of four of the eleven included species were below the detection level. At 60 s and 120 s, there were no CFU counted for seven and eight species. A reduction of ≥3 log_10_ CFU, was found for ten out of the eleven tested species at 30 s (except for *T. forsythia*) and for all species at 60 s. There seemed to be a species-specific effect as four bacterial strains were killed after 30 s of application whereas four were still viable (in reduced counts) after 60 s (Table 1).

### 3.2. Preformed Multi-Species Biofilm on Dentin and Titanium Specimens

The multi-species biofilm was cultivated on two different specimens, dentin and titanium, for 3.5 days. The mean CFU counts of untreated biofilms were 8.16 ± 0.10 log_10_ for those on dentin. On titanium specimens, the respective CFU counts were less (7.76 ± 0.34 log_10_). Colonies typical for oral streptococci (*S. gordonii*) but also for bacteria being associated with periodontal or peri-implant disease (e.g., *P. gingivalis*, *P. intermedia*, *F. nucleatum*, and *T. forsythia*) were always present. The purpose of these experiments was to see if there is activity on an existing biofilm. 

#### 3.2.1. Reduced Biofilm Viability on Dentin Specimens after CAP Application

**Figure 2:**
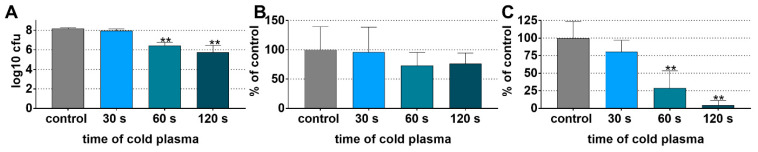
Colony forming units (CFU) counts (**A**), mass (**B**), and metabolic activity (**C**) of multi-species biofilms on dentin specimens and subsequent exposing of 30 s, 60 s, and 120 s to cold plasma. ** *p* < 0.01 vs. control (post-hoc Bonferroni).

Three different analyses were made to quantify the effect of CAP on the biofilm grown on dentin specimens, the total log_10_ CFU, the biofilm mass, and the metabolic activity. The log_10_ CFU/specimen significantly decreased dependent on the time of CAP application. In the mean, the reductions were 0.19 log_10_ CFU/specimen at 30 s, 1.73 log_10_ CFU/specimen at 60 s (*p* < 0.001), and 2.43 log_10_ CFU/specimen at 120 s (*p* < 0.001; Figure 2A). In contrast to the CFU counts, the CAP application did not significantly reduce the biomass which covers the bacteria (viable and non-viable) and the biofilm matrix (Figure 2B). In accordance with the CFU counts, the metabolic activity decreased by about 70% after 60 s of CAP and by 95% after 120 s of CAP (both *p* < 0.001 vs. control; Figure 2C).

#### 3.2.2. Reduced Biofilm Viability on Titanium Specimens after CAP Application

**Figure 3:**
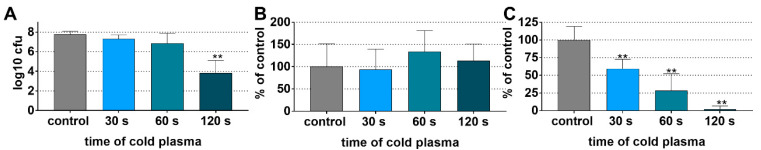
Colony forming units (CFU) counts (**A**), mass (**B**) and metabolic activity (**C**) of a multi-species biofilm on titanium specimens and subsequent exposing 30 s, 60 s and 120 s to cold plasma. ** *p* < 0.01 vs. control (post-hoc Bonferroni).

For the biofilm grown on titanium, the log_10_ CFU/specimen decreased by 0.9 log_10_ after a 60 s application of CAP (not statistically significant) and by about 4 log_10_ CFU/specimen after 120 s of CAP (*p* < 0.001 vs. control; Figure 3A). As on dentin specimens, the CAP application did not have any effect on the biomass (Figure 3B). The metabolic activity of the biofilm decreased in dependence of the CAP application time. There was already a reduction by about 40% after 30 s of CAP (*p* = 0.003), by 70% after 60 s of CAP, and by 98% after 120 s of CAP (both *p* < 0.001 vs. control; Figure 3C). 

### 3.3. No Effect of CAP on De Novo Biofilm Formation

**Figure 4:**
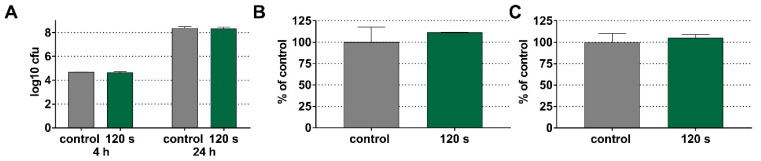
Colony forming units (CFU) counts (**A**) after 4 h and 24 h, mass (**B**), and metabolic activity (**C**) after 24 h of a multi-species biofilm formation on dentin specimens immediately after the application of 120 s of cold plasma.

In the second series of experiments, we answered the question if treatment with CAP may retard a subsequent biofilm formation. Here, only a time of 120 s was used. On the dentin specimens, an application of 120 s of cold plasma did not affect a subsequent biofilm formation. There was never a statistically significant difference visible, neither related to CFU counts, the biofilm mass, nor the metabolic activity in the newly formed biofilm (Figure 4A–C).

**Figure 5:**
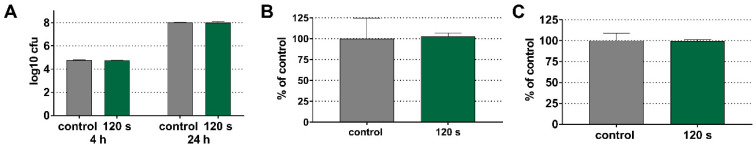
Colony forming units (CFU) counts (**A**) after 4 h and 24 h, mass (**B**), and metabolic activity (**C**) after 24 h of a multi-species biofilm formation on titanium specimens immediately after the application of 120 s of cold plasma.

The same results were found on titanium discs. The biofilm formation was not affected by a 120 s pretreatment of the titanium surfaces and this related to the CFU counts, biofilm mass, and metabolic activity (Figure 5A–C). 

### 3.4. Increased Adhesion of Gingival Fibroblasts to Titanium Surfaces after CAP Pretreatment

**Figure 6:**
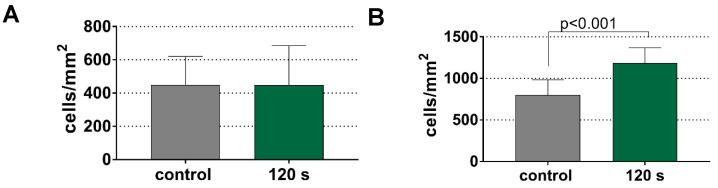
Gingival fibroblast counts per mm^2^ on dentin (**A**) and titanium (**B**) specimens after pretreatment of 120 s of cold plasma.

Ideally, periodontal or peri-implantitis therapy should retard biofilm formation and enhance attachment of host cells, such as oral fibroblasts. The pretreatment of CAP for 120 s did not affect the adhesion of fibroblasts to the dentin surface (Figure 6A). On the titanium surface, the CAP treatment increased the adhesion of gingival fibroblasts to the titanium surface by about 50% (*p* < 0.001 vs. control; Figure 6B). 

### 3.5. No Adverse Effect of Cap on Host Tissue Cells

The major focus of the study was on antibacterial and anti-biofilm activity. However, potential adverse effects should be taken into consideration. Therefore, a few screening assays were performed. Gingival fibroblasts cultured on glass slides were exposed to 10 s, 30 s, 60 s, or 120 s of CAP. The percent of viable cells was in each analyzed sample (four per group) was ≥90%. The mean number of viable cells was between 92.5% ± 2.2% (10 s of CAP) and 93.3% ± 2.1% (60 s of CAP). 

In addition, the temperature on the treated surface was measured. The temperature of the specimens increased in a time-dependent manner. For the dentin specimen, the plasma application resulted in a temperature of 47 °C at 70 s, 50 °C at 92 s, and a maximum of 52.5°C at 120 s. The temperature of the titanium specimen reached 47 °C at 64 s, 50 °C at 114 s, and 50.2 °C at 120 s of CAP application, respectively.

## 4. Discussion

The aim of this study was to investigate the antibacterial and anti-biofilm properties of a piezoelectric CAP device. The device generates plasma from the ambient air and operates in a DBD (dielectric barrier discharge) mode with the treated surfaces acting as the grounded electrode [27]. In terms of the global burden of increasing antibiotic resistance development [36], restricted use of antibiotics is suggested in periodontal and peri-implantitis therapy [37]. CAP may be a promising alternative combining an antimicrobial effect and enhanced tissue healing. 

As a first attempt where the effect of CAP on planktonic single species was analyzed, the activity of CAP was clearly time dependent. An application time of 10 s was not sufficient to reduce the bacterial counts below the detection level in any sample, whereas 70% of the bacteria samples tested negatively after applying 60 s of CAP. The data are in the range observed by others for plasma devices using ambient air. A similar DBD device reduced bacterial counts of methicillin-resistant *S. aureus* (MRSA) by 4 log_10_ within 2 min [38]. In a study using oral planktonic bacteria, the reduction of *S. mutans* and *A. actinomycetemcomitans* was up to 3 log_10_ for both species after 120 s of plasma treatment [39]. A reduction greater than or equal to 3 log_10_ CFU was considered as bactericidal [40]. *p. gingivalis* is the only bacterial species of our experiments that was also investigated by others. In the study by Yang et al. the number of CFU declined by 4.0 log_10_ CFU for 120 s and below the detection level at 6 min of air plasma application [41]. In our trial, *p. gingivalis* was reduced below the detection level after 120 s of CAP application. This finding, and the fact that, except for *T. forsythia,* the counts of all other gram-positive and gram-negative bacteria in the planktonic stage were decreased by at least 3 log_10_ after 30 s of CAP, underline the potential of the used device. 

The results showed some species-specificity. Recently, gram-positive bacteria such as *S. aureus* were found to be less susceptible than gram-negative bacteria such as *Escherichia coli* and *Pseudomonas aeruginosa* [42,43]. In the present study, the antibacterial CAP activity did not differ between gram-positive and gram-negative bacteria. Furthermore, there was no clear dependency on the general tolerance to oxygen for growth. *T. forsythia*, for example, was less susceptible. *T. forsythia* is an anaerobic, gram-negative bacterium with a typical peptidoglycan wall but requires exogenous *N*-acetylmuramic acid for its synthesis [44]. Similar to other oral bacteria, it tolerates a certain degree of oxidative stress, e.g., in biofilm conditions stress proteins are upregulated and resist 20 to 30 times the oxidative stress [45]. *E. corrodens* counts were reduced after 30 s of CAP applications; the viable numbers seemed to remain stable after a longer application. The membrane of *E. corrodens* show a high oxidase activity when being exposed to low concentrations of oxygen [46]. It remains to be clarified if the oxidase activity of the membranes contributes to survival when the bacterium is exposed to CAP. Comparable data are not available. The data of a prototype of the device showed that using the DBD discharge at 2 mm produces mainly NO_2_ and to a lesser extent NO and O_3_. Only traces (<1 ppm) of the other species, including peroxide, were observed [29]. 

A sufficient biofilm eradication is essential for successful periodontal and peri-implant therapy [9,47]. To test an antibiofilm effect, a 12-species biofilm cultivated on dentin or titanium was treated with CAP for different time intervals. Whereas a CAP application of 30 s resulted in a reduction of ≥3 log_10_ CFU for 10 out of the 11 planktonic species tested, there was no difference to the control for the 12-species biofilm at 30 s, but a reduction by 2.4 log_10_ CFU on dentin, and by 3.9 log_10_ CFU on titanium at the maximum application time of 120 s. The maximum time was chosen for being practicable in a clinical situation. Regarding periodontal or peri-implant biofilms, there are only a few reports available. A single-species *A. actinomycetemcomitans* biofilm was exposed to plasma generated from air; the reduction was about 1 log_10_ after 60 s and about 2 log_10_ after 120 s of treatment [39]. Placement of titanium discs with an *A. actinomycetemcomitans* biofilm in an argon plasma chamber for a 12 min cycle killed all bacteria [48]. A 5-day *P. gingivalis* biofilm cultured on titanium discs was treated with an argon plasma jet device; the reduction factor compared to the untreated control was 1.3 log_10_ CFU for 60 s and 180 s application [49]. A six-day biofilm was irradiated with a plasma generated from helium; after 3 min, the reduction was 1.2 log_10_ CFU and after 5 min, no bacteria could be cultured [50]. Within a poly-microbial biofilm, the microorganisms enhance their survival and their resistance to external insults (e.g., antibiotics) by synergistic metabolic interactions, antagonistic interactions, cell-cell signaling, and gene transfer [51,52,53]. Rarely, ex-vivo biofilms were used to study the effect of CAP. An ex-vivo saliva biofilm grown for 48 h on titanium disks in aerobic conditions was treated with different plasma generated from argon in part mixed with O_2_. After 120 s of plasma application, the reduction was up to about 2 log_10_ CFU [54]. The same group of researchers studied the effect of an argon plasma jet against an ex-vivo subgingival biofilm cultured on titanium disks; bacteria were quantified by fluorescent staining, and the intensity of staining was only lowered by 17.4% to 25.2% [55]. In our experiments, the reduction factor of a defined multi-species biofilm cultivated on titanium disks was 3.92 log_10_ CFU after 120 s of CAP application which underlines the good activity. In addition to the CFU counts, the metabolic activity of the biofilm was determined using a resazurin assay [56]. CAP treatment reduced time-dependent metabolic activity of the biofilms cultured on dentin by 96% and titanium by 98%. Other authors found similar results, demonstrating a reduction of 99% in an MTT assay after treating a two-species biofilm (*Streptococcus sanguinis*/*S. mutans*) with argon + O_2_ plasma [57]. 

Complete biofilm removal by using CAP alone does not appear to be possible. No statistically significant reduction in the total biomass was achieved after CAP application neither on dentin nor on titanium, which may underline that the biofilm matrix is not affected by CAP. Our result contrasts with other published data using the same method for determining biofilm mass. An argon plasma jet was as effective in reducing a naturally grown biofilm on extracted teeth as was a sonicated toothbrush [58]. Additionally, the quantity of saliva-biofilms grown on titanium disks and in-vitro at dental implants was reduced by applying CAP [59,60].

In addition to the biofilm removal, a retarding effect on the de novo biofilm formation may be desirable. In the present study, the treatment of the tooth or titanium surfaces did not change the biofilm formation. On one hand, this observation suggests that there was no post-treatment anti-biofilm effect. On the other hand, no enhanced biofilm formation was found, which in turn indicates the absence of any long-lasting negative effects following this approach. The finding on sandblasted titanium disks is similar to that by Kamionka et al. who also did not see a difference in reformation of a subgingival biofilm after 5 days [55]. 

However, adhesion of oral fibroblasts on the treated surfaces is advantageous for the healing process. A pretreatment with CAP increased the adhesion of gingival fibroblasts to titanium surfaces but not to dentin surfaces. The result on titanium is consistent with data for other plasma devices [61,62]. Additionally, a prototype of the same technology (designated for use outside of the oral cavity) showed an enhanced adhesion and proliferation of fibroblasts on titanium due to an increased wettability of the surface [31]. 

Undesired side effects by using CAP in the oral cavity should be avoided. CAP did not show any cytotoxic effect on gingival fibroblasts after direct application. To avoid thermal damage of the surrounding tissue during clinical application, the heat generation should be kept below 50 °C [63]. Therefore, the temperature was measured during a 120 s treatment of dentin, and titanium specimens. The highest temperature measured by infrared thermography were 52.5 °C for dentin, and 50.2 °C for titanium, respectively. In our experiments, the temperature of the dentin specimens was above 50 °C for 28 s. For titanium the temperature exceeded 50 °C for 6 s, respectively. However, it should also be noted that this is below the maximum temperature of 63.6 °C assessed in the oral cavity when drinking black coffee [64]. One critical aspect is the emission of ozone during plasma generation independent of the CAP device. The emitted concentration is 73 mg/h at maximum as published by the manufacturer [27]. Comparable concentrations are generated by in-duct air cleaners and air purifiers [65]. Adequate removal of the excess ozone needs to be ensured, e.g., via sufficient suction similar to ozone delivery devices [66].

Advantages of the methodology of the present studies are the inclusion of bacteria in the planktonic stage and grown as a mixed biofilm on two surfaces (dentin and titanium), and an application time of CAP which might be practicable in the dental practice. As a limitation of the study, it should be noted that flat disks were used, in-vivo, there is a situation of a narrow gap (sulcus) surrounding a tooth or dental implant. Hui et al. created a model simulating a peri-implant lesion and used a spark plasma pen device [60]. In an upcoming investigation, our device will be adapted to the special situation in the oral cavity and respective models established in our laboratory will be used [67]. These models will allow tests on the combination of CAP with a previous mechanical debridement of the biofilm. A few in-vitro studies combined instrumentation with CAP and showed an increased biofilm removal on titanium surfaces [55,59,60,68]. 

The advantages of the used device are the generation of the plasma from air and the proven bactericidal effect on planktonic bacteria and biofilms. The reduction in the bacterial counts in biofilm by using the direct, air-driven device is also comparable to the results obtained with other devices which are already in clinical use in other medical fields, e.g., in dermatology [29,69] or ophthalmology [42]. Further, an adaption of the device with respect to the anatomy of various periodontal areas along with an optimization of the used parameters (i.e., to avoid overheating) are scheduled.

## 5. Conclusions

An air-driven handheld device using the PDD technology and generating a direct plasma from air acts as a bactericidal on planktonic oral bacteria. Further, it shows anti-biofilm activity on multi-species biofilms cultured on dentin and titanium within 120 s of application. These results are promising and underline the potential for implementation in periodontal and peri-implantitis therapy. Further studies are needed to evaluate potential side-effects in more detail and adapt the shape and parameters of the device according to the needs in the dental practice before the efficacy is tested in clinical trials.

## Figures and Tables

**Table 1:** Colony forming units (log_10_; means ± standard deviation) of planktonic bacteria before and after application of cold plasma for 10, 30, 60, and 120 s.

Species	Application Time				
	Control	10 s	30 s	60 s	120 s
*A. naeslundii* ATCC 12104	6.98 ± 0.25	6.72 ± 0.14	0.00 ± 0.00	0.00 ± 0.00	0.00 ± 0.00
*C. gingivalis* ATCC 33624	7.51 ± 0.04	6.93±0.15	2.45 ± 2.83	0.00 ± 0.00	0.00 ± 0.00
*C. rectus* ATCC 33624	7.43 ± 0.04	6.65 ± 0.05	3.52 ± 2.36	0.00 ± 0.00	0.75 ± 1.50
*E. corrodens* ATCC 23834	7.46 ± 0.01	6.64 ± 0.04	1.14 ± 2.28	1.25 ± 2.49	1.71 ± 1.49
*F. alocis* ATCC 33238	7.50 ± 0.02	6.68 ± 0.03	2.33 ± 2.69	1.02 ± 2.03	0.90 ± 1.80
*F. nucleatum* ATCC 25586	6.92 ± 0.24	6.64 ± 0.10	0.00 ± 0.00	0.00 ± 0.00	0.00 ± 0.00
*P. gingivalis* ATCC 33277	7.75 ± 0.06	6.83 ± 0.03	2.59 ± 2.40	1.39 ± 2.06	0.00 ± 0.00
*P. intermedia* ATCC 25611	7.56 ± 0.05	6.64 ± 0.04	0.00 ± 0.00	0.00 ± 0.00	0.00 ± 0.00
*P. micra* ATCC 33270	7.61 ± 0.04	6.79 ± 0.02	0.00 ± 0.00	0.00 ± 0.00	0.00 ± 0.00
*S. gordonii* ATCC 10558	7.54 ± 0.08	6.40 ± 0.54	3.13 ± 2.02	0.00 ± 0.00	0.00 ± 0.00
*T. forsythia* ATCC 43037	6.93 ± 0.05	6.71 ± 0.03	6.73 ± 0.01	3.38 ± 3.90	0.00 ± 0.00

## Data Availability

Data is contained within the article.
